# AGING BODIES IN PARADISE? AN ETHNOGRAPHIC STUDY OF FANTASY FEST IN KEY WEST, FLORIDA

**DOI:** 10.1093/geroni/igz038.1715

**Published:** 2019-11-08

**Authors:** Rachel A Douglas, Anne Barrett

**Affiliations:** Florida State University, Tallahassee, Florida, United States

## Abstract

Dominant cultural constructions of aging bodies, particularly those of women, as unattractive and asexual may be challenged within politically and socially progressive leisure environments, like Key West, Florida, that promote out-group acceptance, collectivity, and cultural diversity. However, this possibility receives limited scholarly attention. Addressing this gap, our study employs observational and interview data (n=60) collected in 2017 and 2018 at Key West’s Fantasy Fest – an annual event marketed as a “10-day party in paradise for grown-ups.” The festival, drawing as many as 100,000 people, cultivates a relaxed atmosphere permissive of nudity and theatrical body adornment, including body paint and costume. This feature makes it an ideal site for examining the effect of inequalities, including age and gender, on body displays and social reactions to them. Data analysis revealed four themes centering on aging bodies – Judging Bodies, Limiting Body Displays, Displaying Bodily Difference, and Liberating Bodies. Age and gender inequality strongly influenced judgments of attractiveness and sexual appeal, contributing to older participants’ more limited body displays. Nevertheless, both young and old participants collectively contributed to creating a liberating environment that celebrates bodily difference and encourages cross-age interaction. While limited to one site with a unique political and social climate, our study suggests the potential of progressive leisure environments to broaden notions of aging bodies and encourage cross-age connections.

